# Phase partitioning during fragmentation revealed by QEMSCAN Particle Mineralogical Analysis of volcanic ash

**DOI:** 10.1038/s41598-018-36857-4

**Published:** 2019-01-15

**Authors:** A. J. Hornby, Y. Lavallée, J. E. Kendrick, G. Rollinson, A. R. Butcher, S. Clesham, U. Kueppers, C. Cimarelli, G. Chigna

**Affiliations:** 1 Department of Earth, Ocean and Ecological Sciences, University of Liverpool, 4 Brownlow Street, Liverpool, L69 3GP UK; 2 Camborne School of Mines, CEMPS, University of Exeter, Penryn Campus, Treliever Road, Penryn, Cornwall, TR10 9FE UK; 3 Geological Survey of Finland, Espoo, FI-02151 Finland; 4 Department of Earth and Environmental Sciences, Ludwig-Maximilians-Universität München, Theresienstrasse 41/III, 80333 Munich, Germany; 5 Instituto Nacional de Sismologia, Vulcanologia, Meteorologia e Hydrologia (INSIVUMEH), 7a Avenue 14-57, Zone 13, Guatemala City, Guatemala

## Abstract

Volcanic ash particle properties depend upon their genetic fragmentation processes. Here, we introduce QEMSCAN Particle Mineralogical Analysis (PMA) to quantify the phase distribution in ash samples collected during activity at Santiaguito, Guatemala and assess the fragmentation mechanisms. Volcanic ash from a vulcanian explosion and from a pyroclastic density current resulting from a dome collapse were selected. The ash particles resulting from both fragmentation modes are dense and blocky, typical of open-vent dome volcanoes and have a componentry consistent with their andesitic composition. We use image analysis to compare the fraction of each phase at particle boundaries compared to the total particle fraction. Our results show that the explosion-derived ash has an even distribution of plagioclase and glass, but boundaries enriched in pyroxene and amphibole. In contrast, the ash generated during dome collapse has an increased fraction of glass and decreased fraction of plagioclase at particle boundaries, suggesting that fractures preferentially propagate through glass during abrasion and milling in pyroclastic flows. This study presents QEMSCAN PMA as a new resource to identify generation mechanisms of volcanic ash, which is pertinent to volcanology, aviation, respiratory health and environmental hazards, and highlights the need for further experimental constraints on the fragmentation mechanism fingerprint.

## Introduction

### Background

Volcanic ash, as the product of fragmentation, is a near-ubiquitous phenomena at active volcanoes^1^, irrespective of the failure modes and mechanisms at play across a broad spectrum of eruptive activity. Primary fragmentation mechanisms produce ash directly from magma within volcanoes: through gas overpressure fragmenting foamed magma^2^, interaction between magma and water^3^, or through strain-induced shear failure at conduit boundaries^4^. Secondary fragmentation processes occur via particle interactions in pyroclast-laden flows^5^ and during slip in fault gouge^6^, and can modify existing particles and create new ash-sized particles^7,8^. Secondary fragmentation processes include particles produced by milling^9^, abrasion^5,10,11^ and attrition^6,11^. Studies have shown that different eruption styles and intensities modify particle properties^12–14^. The size, composition, dispersal and sedimentation of ash contribute to the degree and nature of hazards to respiratory health, the natural environment, infrastructure and aviation^15,16^, and of benefits to the biosphere^17–20^.

The origin of volcanic ash is often categorised simply by comparison to eruptive behaviour or ash particle geometry. For instance, the ash of large Plinian eruptions (which is commonly crystal poor) has been characterised to geometrically represent the glassy junctions of bubble walls accompanying magmatic fragmentation by pore overpressure^21^. On the other hand, weak to moderate explosions at lava dome volcanoes, which are frequently understood as the result of the disruption of a relatively impermeable, dense magma plug by an underlying bubbly magma^22^, produces ash particles with variable shapes, including dense, blocky fragments and porous fragments that contain irregularly shaped pores^23^. In cases where lava domes develop marginal shear zones in the shallow conduit, such as during the 2004–2008 eruption at Mount St. Helens, studies have noted similarities (in shapes and chemistry) between the ash retrieved from plumes and those recovered from the fault gouge along spine margins^24,25^. This has led to the conclusion that ash produced during the extrusion of dense spines was sourced primarily from faulting, fracturing and abrasion of a high viscosity magma plug^23^. Failure and wear mechanisms are of particular importance in dense, highly viscous dome lavas, where brittle deformation and slip in areas of strain localisation can regulate eruptive behaviour at shallow depths^24,26,27^. At the Guatemalan volcanic complex Santiaguito (the site of this study), frictional heat generated during shallow faulting events has been proposed as a contributor to late-stage vesiculation that triggers fragmentation and explosion^27^; a mechanism that can be identified by the presence of chemically heterogeneous melt patches resulting from selective melting of individual crystal phases. Thus the properties of volcanic ash may reflect a combination of processes and a genetic description remains to be obtained to advance our understanding of shallow magmatic processes associated with different eruptive scenarios.

Only a handful of studies have investigated the abundance or distribution of mineral and glass phases within ash particles with respect to fragmentation mechanisms. Automated scanning electron microscopy with energy dispersive X-ray spectrometry (SEM-EDS) mapping was used to evaluate the phase and size distribution in ash retrieved from a Boeing 747 following the eruption of Redoubt^28^. The important role of plagioclase feldspar in intermediate-silicic magmas has been identified, showing that the characteristic size of plagioclase feldspar crystals modified the grain size distribution of airfall ash^29^. At Soufrière Hills Volcano, Montserrat, an increased fraction of plagioclase feldspar was found in vulcanian (plume-derived) ash compared to flow-derived ashfall deposits^14^, which was attributed to segregation of coarser and denser crystal-rich particles within pyroclastic density current (PDC) deposits^30,31^. In addition, it has been recognised that abrasion is phase-selective, favouring fracturing and chipping of glass, therefore it has been proposed that the degree of abrasion can be estimated by the thickness of glass rims surrounding phenocrysts in ash particles^7^. A recent experimental comparison of particle characteristics provided data on particle attributes during primary (decompression) fragmentation, milling and collision, revealing characteristic variations in glass rim thickness and crystal size and highlighting distinctive changes in the fractal dimension of the size distribution^11,32^. Finally, the presence and relative abundance of interstitial glass requires consideration with regard to kinetic processes and fracture mechanisms in ascending magmas^33,34^. For instance, changes in ash particle componentry and glass chemistry have been used to infer variations in magma ascent rate that determine eruptive behavior^35^. The petrological study of volcanic ash has provided a wide range of proxies to understand certain magmatic (mechanical) processes, yet, integrated physicochemical descriptions are required to fully understand the controls on and impact of ash properties on volcanic processes and hazards.

In recent years, there have been growing efforts to record particle properties produced by different fragmentation processes and relate these to hazard mitigation, risk management and forecasting efforts^36^. Here, we use a powerful automated mineralogy tool, QEMSCAN Particle Mineralogical Analysis, and develop a simple image analysis routine to describe the changes in phase distribution in particles produced during vulcanian explosions and dome collapse events at Santiaguito in Guatemala.

### Santiaguito dome complex, Guatemala

**Figure 1:**
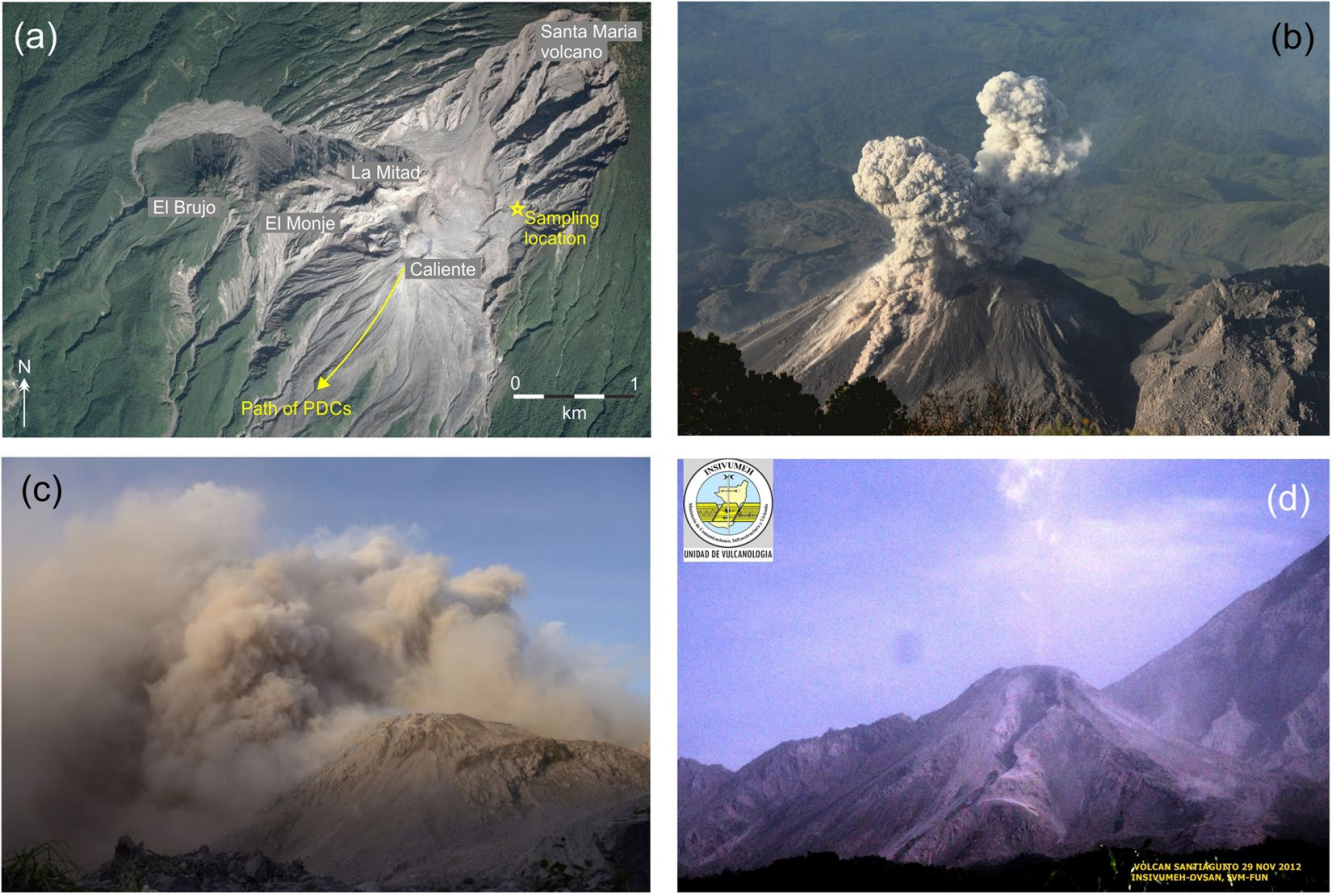
(**a**) Satellite imagery showing the Santiaguito dome complex and Santa Maria volcano, with the sampling location and PDC path for the dome collapse event in panels c-d shown. Map data: Google Earth, CNES/Airbus, DigitalGlobe. (**b**) The Caliente vent at the Santiaguito dome complex in Guatemala, showing a typical vulcanian explosion from 9^th^ November 2012 including a small pyroclastic flow and the separation of gas- and ash-rich plumes at the onset of explosion. Ash sample from a similar vulcanian explosion (referred to as VE in this study) was collected from a similar event on 13 November 2012. (**c**) The co-pyroclastic ash cloud following a partial dome collapse on the southern flank of the Caliente vent on 28 November 2012; photo taken form the Paradiso camp 600 m from the vent. An ash sample from this dome collapse event (referred to as DC in this study) was collected 90 minutes after the photo was taken. (**d**) Photograph from Santiaguito volcano observatory (INSIVUMEH/OVSAN) on 29 November 2012 looking northward and showing the aftermath of the dome collapse event. A steep-sided gulley was eroded into the flank by the collapse and the passage of pyroclastic flows, which progressed for >5 km in a southerly direction.

The Santiaguito volcanic complex, in Guatemala, consists of a chain of four dacitic lava domes formed along a fracture at the base of a sector-collapse scarp associated with the 1902 Plinian eruption of Santa Maria stratovolcano^37–39^ (Fig. 1a). The Santiaguito dome complex has been continuously active since the initiation of dome-building in 1922. The Caliente dome is the earliest active structure as well as the only vent that has shown near-continuous discharge of lava interspersed by regular explosions throughout the eruptive history. Today, the regular generation of gas-and-ash clouds (see Fig. 1b) and the episodic occurrence of PDCs associated with explosions and dome collapse events are the greatest hazards to surrounding communities farming the flanks of the volcano. Partial dome and flow collapse events have generated many of the most destructive PDCs in recent years, reaching nearby communities and plantations (e.g., October 7–10 2003, where 250 acres of coffee plantation was destroyed; November 28 2012, which deposited 2 cm of ash on local communities (Fig. 1c,d); May 9 2014, a large collapse generated PDCs reaching 7.5 km and causing substantial economic losses in a nearby plantation and leading to the evacuation of 1000 people – Pers. Communication, Julio Cornejo, Santiaguito Volcano Observatory (OVSAN)/National Institute for Seismology, Volcanology, Meteorology and Hydrology of Guatemala (INSIVUMEH)). These events produce abundant airborne ash particles as seen in photos from the November 28 2012 event shown in Fig. 1c. The weak vulcanian explosions (volcanic explosivity index (VEI) 0–2) typically occur at regular intervals of 20–120 minutes and produce gas-rich emissions followed by discreet ash-rich bursts (Fig. 1b), which evolve into small (500–1000 m) ash-poor plumes^40,41^. The explosive products typically erupt along arcuate fractures, some radial, but most concentric across the 200 m wide dome surface^42^.

Here, we characterise volcanic ash from a typical vulcanian explosion and a dome collapse event to compare the mechanisms and products of fragmentation processes at this incessantly active dome-building volcano.

## Materials and Methods

### Ash collection

Ash samples were collected 15 days apart at the same location, ~500 m northeast from the active Caliente vent (Fig. 1a) during a field campaign in November 2012. The ash samples were collected by laying clean canvas sheets on the ground prior to eruptive activity. After ash settling, we used a paintbrush to collect the particles into sealable sample containers. The sheets were carefully cleaned between each collection, providing ash samples linked to individual eruptive events. This study compares two samples: (i) collected following a single deposition event on 13 November, during typical gas-and-ash venting associated with vulcanian activity (see Fig. 1b); (ii) collected at 09:00 on 28 November following deposition from an ash cloud (Fig. 1c) generated by the collapse of a lava lobe that had breached the south-western rim of the Caliente dome and advanced approximately 100 m down the south-facing flank. The Guatemalan monitoring agency, INSIVUMEH, reported stronger-than-usual pyroclastic flows on the southern flanks of Caliente during this time, generating ash clouds that rose 3.5 km and deposited ash up to 70 km from the vent (Fig. 1c). Visibility at the collection site was reduced to ca. 10 m during the heaviest ashfall and the canvas sheet was covered by ~5 mm of ash. The Caliente vent was obscured by airborne ash for approximately six hours. The following morning, a deep erosional channel could be seen in the southwestern flank from OVSAN (Fig. 1d), marking the path for PDCs following the dome collapse. In the 48 hours before and after this event, the Caliente dome had a period of explosive quiescence, with no explosions witnessed, in contrast to the regular gas and ash explosions. The effusion rate was historically high, with four active lava flows on the south flanks, and the lava lobe involved in the dome collapse was replaced in 72 hours (Julio Cornejo, personal communication). These two samples, i) deposited following a vulcanian explosion (termed hereafter sample VE) and ii) following a partial dome collapse (hereafter sample DC) respectively, are used to compare the characteristics of volcanic airfall ash generated by vulcanian explosions and dome collapse events at Santiaguito.

### QEMSCAN Particle Mineralogical Analysis (PMA)

Textural and mineralogical analysis of the volcanic ash samples was conducted using scanning electron microscope photomicrographs and phase images acquired by QEMSCAN. QEMSCAN (Quantitative Evaluation of Minerals by Scanning Electron Microscopy) is the brand name for an automated SEM-EDS mapping system for compositional mineral/phase mapping. Originally designed in the late 1970 s for the mining industry^43^, it has gone through extensive development before commercial release in its current form in the early 2000s. Since then, it has been used for research purposes and has been successfully applied to many types of projects and samples^43–48^. The system used in the study was a QEMSCAN 4300 installed at Camborne School of Mines, and consists of a Carl Zeiss Evo 50 SEM with four light element Bruker Xflash Silicon Drift energy dispersive X-ray detectors. The system employs a combination of back scattered electron (BSE) intensity and low-count EDS X-ray data to raster a sample area with a micron-scale step size, producing thousands to millions of chemical analysis points in a single map^47^. The iDiscover software suite uses each analysis point to classify a mineral or phase from the chemical elemental data by comparing against a species identification protocol (SIP) database that can be customised to characterise a sample using *a priori* compositional data. The final output is a bitmap image where each pixel represents a measurement point. In PMA mode, the software can characterise the size and shape of the particles in addition to the constituent components. The QEMSCAN technique enables the rapid and automated mapping of geological samples on a micron scale to provide superior data of many sample types, but the scan is limited to a ~3% elemental detection limit per analysis point due to the rapid speed of operation, and other documented SEM-EDS limitations^48^.

**Figure 2:**
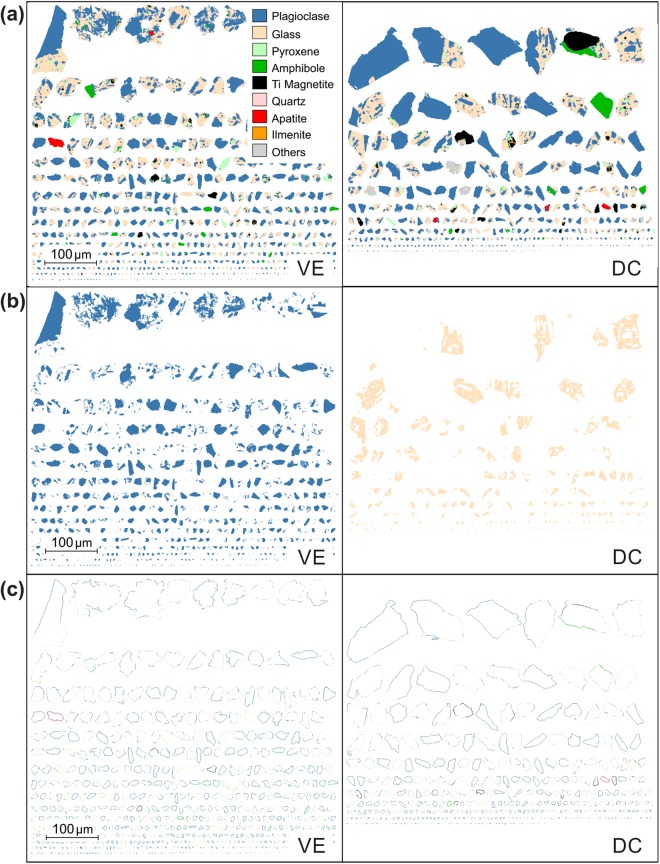
1 μm resolution QEMSCAN PMA maps used in image analysis for the vulcanian explosion (VE) sample (left) and partial dome collapse (DC) sample (right). (**a**) Original QEMSCAN PMA image showing phase distribution within ash particles; (**b**) Single-phase (isolated) plagioclase feldspar image from sample VE and interstitial glass (sample DC) within the particle population (see methods). (**c**) Ash particle boundaries. In all images, the particles are sorted by size. The key shows the mineral or phase species identified during QEMSCAN analysis (details shown in Table S1). The key and scale correspond to all panels.

For this study, QEMSCAN measurements in PMA mode were conducted on polished thin section surfaces at resolutions of 1 and 2 μm for unsieved aliquots of each sample. The automated analysis results were manually checked by comparison with a 1 μm resolution map that contained chemical elemental and phase classification data for ~300 particles from each ash sample. Eleven phases were assigned with criteria weighting in the order given for each phase in Supplementary Table 1, available online. The phase assignment was completed manually by addition of the interstitial glass composition, determined by published electron probe microanalysis^49^, to the SIP database, and by consolidating similar mineral compositions from solid solution series (i.e. plagioclase feldspar and pyroxene) under a single classification. We produced one particle-size sorted PMA image at each resolution to create two images per ash sample. The images comprised 621 and 2141 ash particles for sample VE, and 408 and 1444 particles for sample DC, at 1 μm and 2 μm resolution respectively. Each image was output as a 12 colour, 8-bit tiff file (e.g. Fig. 2a,b; full-resolution images available online in Supplementary Figs 1–4). In order to assess the size-dependent variation in phase distribution, each image was used to produce 11 single-phase images by colour thresholding to separate each phase (see Fig. 2c,d).

### QEMSCAN phase fraction and distribution

Particle boundary images were also produced from the original QEMSCAN tiff files by thresholding to binary, filling any holes, and running an internal gradient filter with 1 pixel resolution and a diamond-shaped structuring element using the MorphLibJ plugin^50^, freely available from http://imagej.net/MorphoLibJ. The structuring element is a ‘probe’ shape used to perform morphological operations on test shapes. For example, a dilation operation preserves the structuring element everywhere it overlaps a pixel containing the test shape, enlarging the test shape, while an erosion performs the opposite function. The ‘morphological gradient’ operation is the difference between the result of a morphological dilation and a morphological erosion, defining the boundary of the test shape. At its smallest size, the diamond-shaped structuring element must be centred 1 pixel distance from a test shape to intersect it, therefore, it produces a 1-pixel-width boundary during a morphological gradient. The binary boundary image was then merged to the original tiff image using the Image Calculator ‘AND’ function in ImageJ to produce a full-colour boundary image. The boundary images were then separated into single-phase images by colour thresholding. The number of pixels of each colour (both for full particle and particle boundary images) were counted using the Colour Inspector 3D plugin for ImageJ (freely available from https://imagej.nih.gov/ij/plugins/color-inspector.html) to compare phase fractions. The weighted percentage from 1 µm and 2 µm resolution images were added to give the total fractions for each sample. In order to calculate the size-dependent phase distribution, particle boundaries were first saved to the region of interest (ROI) manager. An image stack containing the single-phase images was opened, converted to binary and scaled. The Multi-Measure function within the ROI manager was then used to measure the area of each phase within each ROI. This process was run on both full particle and particle boundary images. Minimum phase sizes of 20 µm^2^ for 1 µm resolution images and 100 µm^2^ for 2 µm resolution images were chosen for analysis. The total number of particles analysed was 2181 for sample VE and 1722 for sample DC. The area equivalent circle diameter, *D*, was calculated for each particle and converted to Phi units, where ${\rm{\Phi }}=-{{\rm{l}}{\rm{o}}{\rm{g}}}_{2}D$. The area fraction of each phase was calculated for each particle, and the average phase fraction calculated within size bins ranging from 2.5–7.5 Ф (equivalent to circular diameters of approx. 6.6–210 µm).

## Results

### Petrographic description of volcanic ash

**Figure 3:**
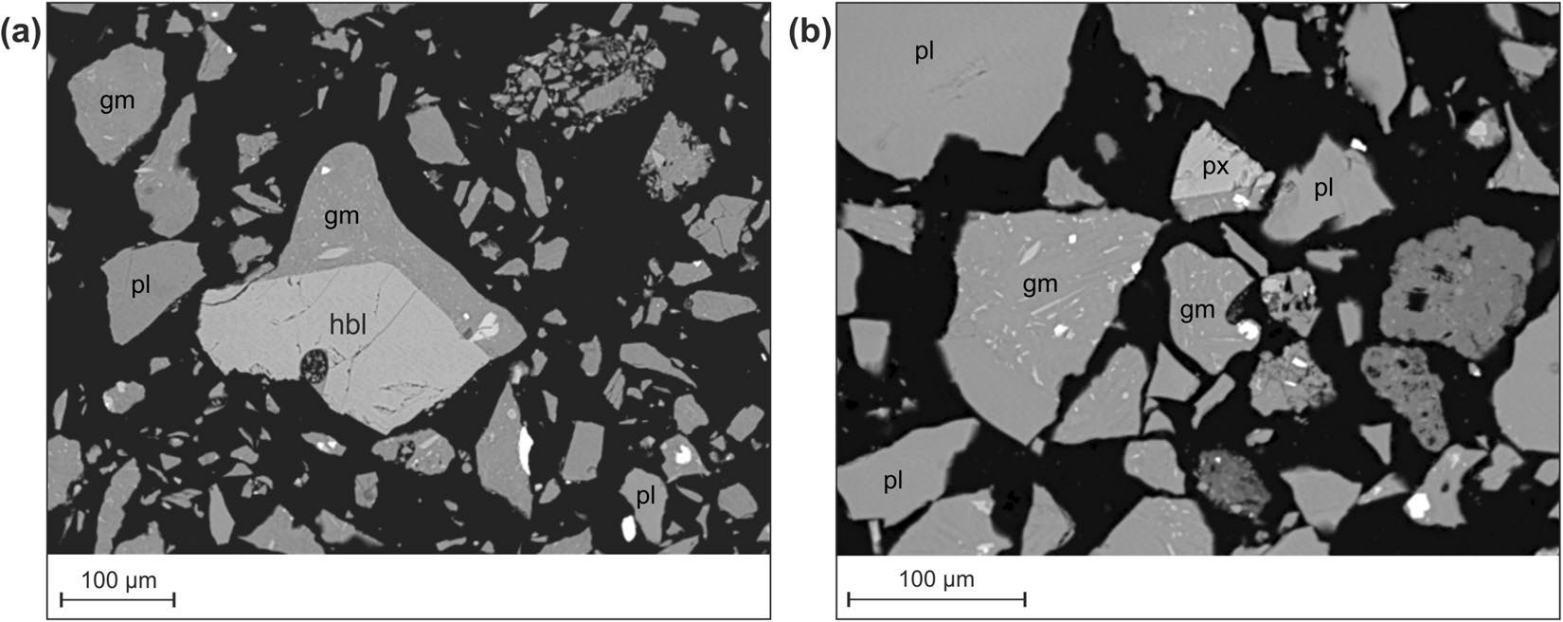
(**a**) Typical ash particles from sample VE, generated by vulcanian activity at Santiaguito. Note the blocky, dense particle shapes and the lack of vesicles. pl = plagioclase, px = pyroxene, gm = groundmass (glass and microlites) hbl = amphibole (hornblende). (**b**) Blocky ash particle from sample DC showing partial vesicularity in some particles, suggesting heterogeneous bubble textures. Note in both samples the lack of concavities that would be associated with fracture propagation between bubbles, commonly used to indicate magmatic fragmentation.

Volcanic ash erupted at Santiaguito is made up of phenocrysts of plagioclase, pyroxene, amphibole and titanomagnetite (Ti-magnetite) in a groundmass of dacitic-rhyolitic (66–78% SiO_2_) glass^49^ hosting plagioclase microlites and minor oxides (Fig. 3). The bulk chemistry was measured by x-ray fluorescence, and demonstrates a dacitic host magma, with SiO_2_ around 60% (Table 1). The microlites are tabular and typically 5–10 μm in length. Large plagioclase phenocrysts are common, and ash particles may be wholly composed of crystal fragments, consist entirely of groundmass glass and microlites or commonly contain a mixture. BSE images taken on an SEM show a range of petrological textures. Qualitatively, most particles appear dense, blocky and angular with low aspect ratio and no evidence for smooth concave margins, which would indicate the pre-fragmentation presence of large vesicles. Vesicles are rare and are generally small (<30 μm) with a low aspect ratio, and not necessarily distributed along particle boundaries.

**Table 1:** Major elements (in wt. %) for ash samples VE and DC measured by X-ray fluorescence.

Sample	SiO_2_	TiO_2_	Al_2_O_3_	Fe_2_O_3_	MnO	MgO	CaO	Na_2_O	K2O	P_2_O_5_	SO_3_	*LOI	Total
VE	60.94	0.52	18.85	4.70	0.115	1.83	6.25	4.77	1.476	0.200	0.038	0.45	100.14
DC	60.57	0.46	19.35	4.21	0.103	1.38	5.83	4.47	1.348	0.205	0.036	0.90	98.85

*LOI = Loss on ignition.

### Phase fractions and distribution

**Figure 4:**
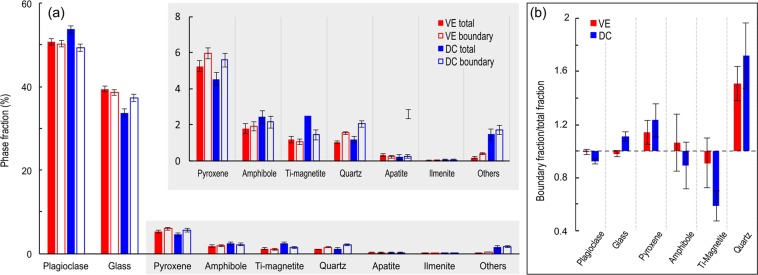
(**a**) The average fraction of each phase in all ash particles analysed for samples VE (red bars) and DC (blue bars). Solid bars show the total phase fraction and open bars show the boundary phase fraction. Inset: Magnified view of phases with <6% area fraction, highlighted in grey. The key in the top right applies to both the main panel and the inset. (**b**) The ratio between phase fraction at the boundary to the total phase fraction for the six most abundant phases in samples VE (red bars) and DC (blue bars).

**Figure 5:**
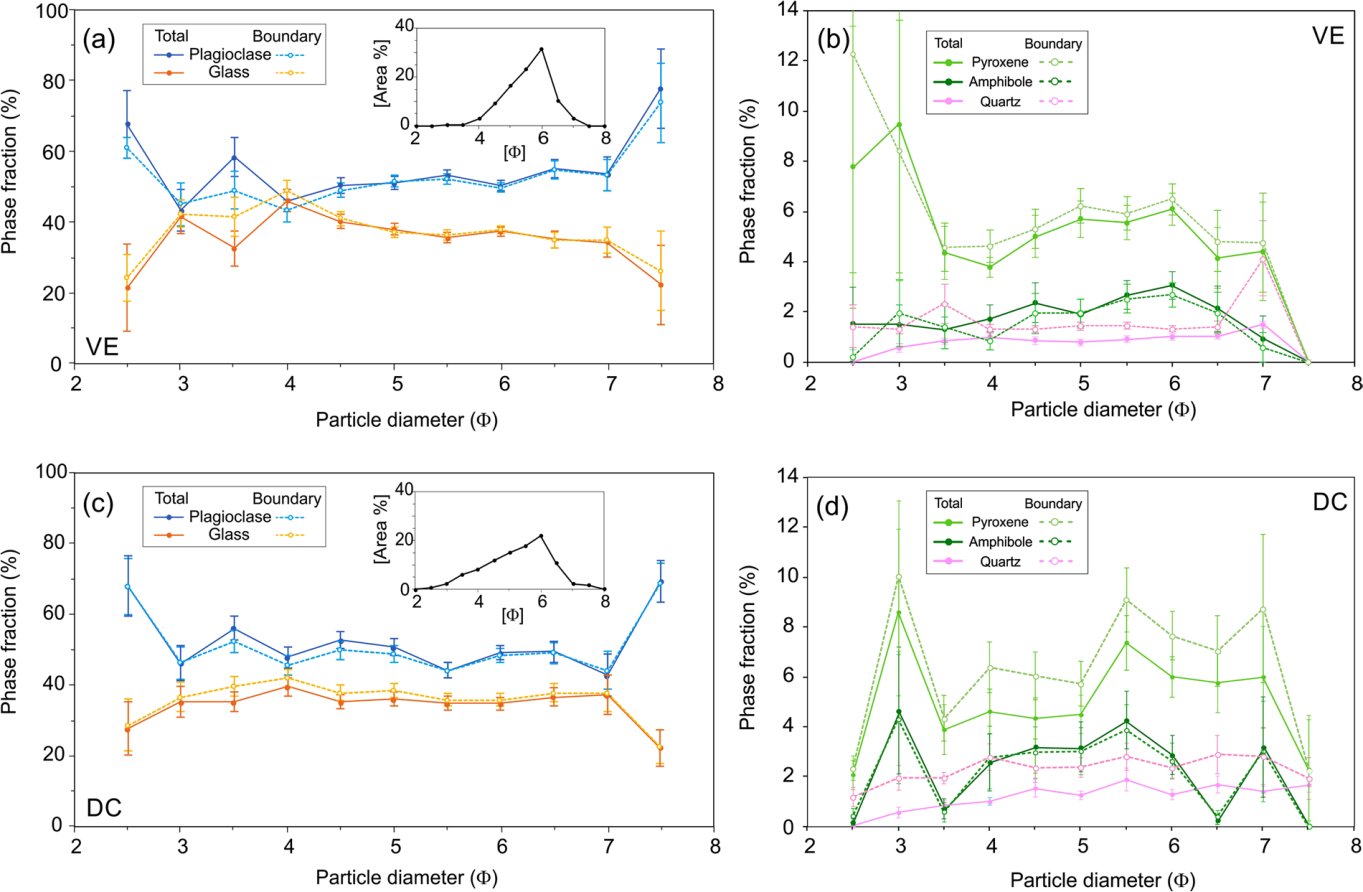
Variations in the total fraction and boundary fraction (%) of 5 major phases plotted against particle area for samples VE (**a**,**b**) and DC (**c**,**d**) taken from all particles in discrete sized bins (11 bins from 2.5–7.5 Ф). The standard error of the mean is shown for each data point. Note the change in scale for the y-axis in panels (**b**,**d**). The grain size distribution of the particles in the analysis are shown as insets for samples VE in panel (**a**) and DC in panel (**c**).

The average fraction of each phase in the ash particles as well as the average fraction in the single-pixel boundary of every ash particle have been quantified for both ash samples and are presented in Table 2. It should be noted that ash particle componentry consists of approx. 90% plagioclase (phenocrysts and microlites) and groundmass glass. Mineral/ phase assignment criteria agree well with a previous detailed description^49^, however identification of some phases remains ambiguous, such as ‘Glass 2 (Al, Si, O phase distinct from the primary glass)’, ‘Fe Al silicates’ and ‘Others’. Here we exclude these phases from analysis and interpretation as they altogether comprise <2% of the total mineralogy. The average distribution of phases within the ash particles comprising samples VE and DC are shown by plotting the total area fraction and the boundary fraction for each phase (Fig. 4a). The ratio of the average boundary fraction relative to the total fraction for the six most abundant phases is highlighted in Fig. 4(b), where a value of 1 shows identical boundary and bulk phase fraction, while higher and lower values indicate enrichment or depletion of the phase at particle boundaries, respectively. At particle boundaries, sample DC contains on average 37.3% glass while the average total glass fraction is 33.8%, giving an increase in glass by a factor of 1.1 at particle boundaries, which is matched by a similar depletion by a factor of 0.92 in plagioclase across all particles (Fig. 4b). In contrast, the vulcanian explosion sample (VE) shows <1% variation between whole particle and boundary distribution of all phases (Table 2), with only minor relative changes in plagioclase and groundmass glass fractions at particle boundaries (Fig. 4b). Significant enrichment of pyroxene at particle boundaries relative to the bulk fraction, by a factor of 1.13 for sample VE and 1.23 for DC), while amphibole is enriched in sample VE but depleted in sample DC, however these values have significant associated error. Sample DC is significantly more depleted in Ti-magnetite and enriched in quartz at particle boundaries than sample VE. Thus phase distribution appears distinct in different ash generation mechanisms. We constrain the size-dependence of phase variations at particle boundaries by measuring the phase fraction and boundary phase fraction for every ash particle and comparing the average within particle size distribution (PSD), binned between 2.5–7.5 Ф (Fig. 5). Total fraction and boundary fraction of plagioclase and glass are covariant and show relatively minor size dependence.

**Table 2:** The average total phase fraction (labelled Total) and the average phase fraction at the 1 µm particle boundary (labelled Boundary) in percent for all particles in ash samples VE and DC.

Phase	VE		DC	
	Total	Boundary	Total	Boundary
Plagioclase	50.76	50.28	53.73	49.43
Glass	39.43	38.56	33.80	37.31
Pyroxene	5.24	5.96	4.55	5.59
Amph	1.79	1.90	2.43	2.15
Ti Mag	1.17	1.07	2.49	1.45
Quartz	1.03	1.56	1.20	2.06
Apatite	0.34	0.23	0.24	0.25
Ilmenite	0.04	0.02	0.08	0.05
Others	0.18	0.42	1.48	1.71

Sample VE shows relatively constant plagioclase and glass fractions as a function of particle size, and the largest variations at grain sizes larger than 4 Ф can be attributed to single particle variations as few particles are present in this size range (see inset in Fig. 5a). For the dominant grain sizes (3.5–6.5 Ф), we observe negligible differences in the distribution of plagioclase and glass at the particle boundaries. In contrast, finer particles appear depleted in amphibole and enriched in plagioclase, while quartz enrichment is found across the size range (Fig. 5). The greatest absolute differences in phase fraction between the ash samples are seen in glass and plagioclase (Fig. 4) for sample DC and these are plotted in Fig. 5c; glass appears to be preferentially distributed (at the expense of plagioclase) along the boundary, particularly within the coarser grain sizes (3.5–5 Ф); these values all fall within error, however the overall distribution shows an enrichment at particle boundaries that is outside of error (Fig. 4). Enrichment of quartz and apparent enrichment of pyroxene at particle boundaries is found for all grain sizes, while amphibole shows little variation.

**Figure 6:**
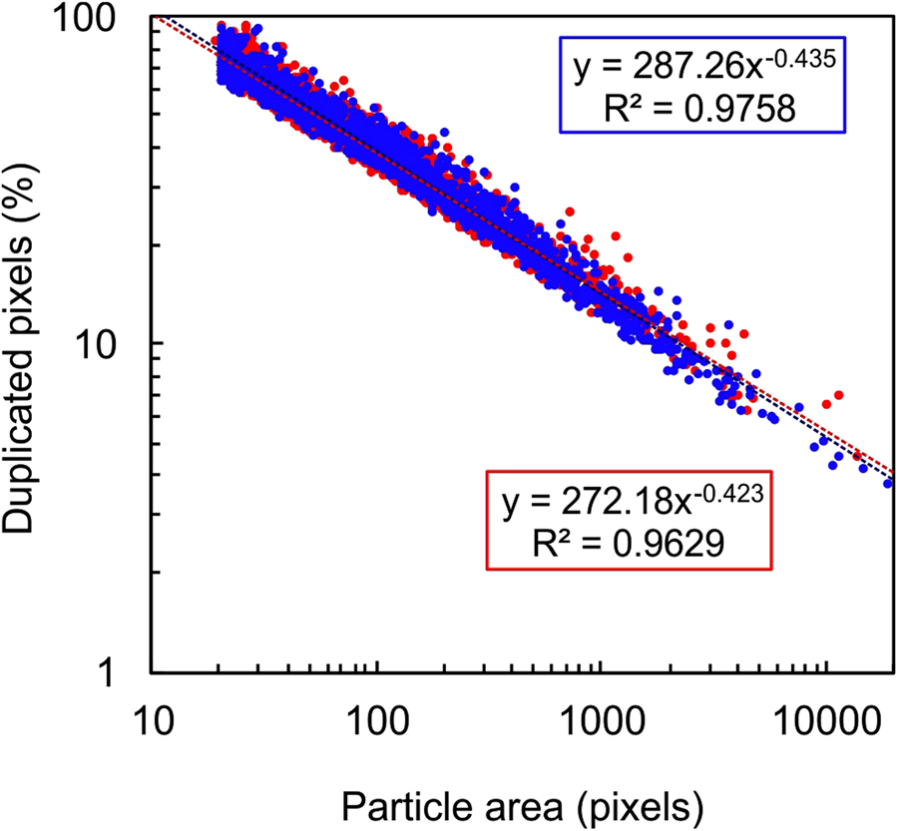
The percentage of the boundary pixel area that is duplicated in total pixel area against particle size for all particles used in Fig. 5 (VE and DC shown in red and blue dots respectively). Both samples show similar trends, indicating only small differences in the shape and vesicularity of constituent particles.

It may be observed in Fig. 5(b) that the boundary and bulk phase fractions converge at smaller particle sizes: this is an artefact of the increasing proportion of the single-pixel boundary to the total particle size. The ratio of boundary to total pixels increases with decreasing particle size and circularity and increasing porosity. In our results the fraction of the pixels in the particle boundary to the total follow a monomial power law for both samples as shown in Fig. 6. These results show that the phase variation results in Figs 4(b) and 5 are minimum estimates, particularly at smaller grain sizes. The particle size distributions shown in Fig. 5 are similar to PSD measured using laser particle size analysis on the ash samples prior to mounting (Supplementary Fig. 5, available online). Particle size estimation from 2D projections produced by QEMSCAN have been shown to underestimate the true grain size^51^ and therefore the modal peak and the mean of the PSDs shown in Fig. 5 would be shifted to coarser sizes, bringing them closer to the values found using laser diffraction spectroscopy (Table S2).

## Interpretation and Discussion

### Mineralogical controls on fragmentation

Imaging of the phase distribution within the volcanic ash particles developed during contrasting eruption styles can provide one of the most striking constraints on the fragmentation processes. Discrimination between ash produced by magmatic fragmentation and attrition processes can be found in the distribution of phases in the ash and at the particle boundaries (Figs 4–5). Here, variation in the distribution of all major phases for sample DC, and pyroxene, amphibole and quartz for sample VE suggests that fracture locations during natural fragmentation of multi-phase volcanic material are not randomly distributed, implying that fragmentation mechanisms may induce physico-chemical differentiation of ash particle surfaces, as suggested by a recent surface reactivity studiy^52^. An understanding of the influence of a mineralogical assemblage on the range of fragmentation processes involved in volcanic eruptions remains limited to date, whereas a substantial body of research has experimentally investigated the roles of porosity, permeability, viscosity, microstructure and stress conditions on the rupture of volcanic rocks^53–55^ and lavas^56,57^. Undoubtedly, physical heterogeneities in the host rock or magma at Santiaguito, for example pores and microfractures, exert a fundamental control on the development of faults and fractures^58–60^, however at Santiaguito, the porosity of the ash particles and the fraction of concave particle shapes are very low (Fig. 3), suggesting that pore pressure and pore-emanated cracking may have played a minor role in the fragmentation process. This may indicate that microfractures efficiently propagate and coalescence, thus exerting a great influence on fragmentation in dense dome lavas. A thorough evaluation on the role of microstructures on the generation of natural ash samples, which highlights the importance of the constituent phases on fragmentation, is beyond the scope of this study but remains an important research question particularly for numerical modelling of fragmentation^61^.

It has long been known that rock-forming minerals have different strength and hardness^62,63^; therefore their response to an imposed stress field is believed to vary. The problem of predicting fracture locations with regard to phases during rock fragmentation has been studied in depth by the mineral engineering industry^64,65^ in order to maximise the profitability of ore processing. A key goal of the industry is to encourage phase boundary fracturing, leaving crystal faces exposed at the boundaries of comminuted clasts^46^. In tephra, the fraction of liberated crystals may correlate with the fragmentation energy^66^; however explosive energy has also been correlated to intra-crystal fractures^67^ and the particular response may depend upon componentry and texture. Indeed, the initial texture and crystallinity of a magma may be a determining factor for the fragmentation mechanism(s) at work^68^ and the fragmentation efficiency^69^.

In some stress regimes, glasses (and melt phases subjected to stress favouring a brittle response) are generally stronger than individual crystals or crystalline materials; yet, both the strength of glasses and crystal-rich rocks further increases with temperature^70,71^, although their relative strengthening remains to be characterised^56^. Previous work has demonstrated that attrition during milling preferentially abrades glass around crystals, leaving a glass-enriched margin^7,32^, rounding of glass-rich particles and an increased fraction of glassy fines in elutriated ash^9,14^ due to density-controlled segregation^14,30,31^. Thus, our observation that particles are enriched in glass and depleted in plagioclase at the boundary in sample DC (Fig. 4) indicates likely processing through milling and abrasion in PDCs, as the major phenocryst phase is enclosed in a glass-enriched margin. The fact that we find a lower total glass fraction in sample DC compared to VE may result from a combined contribution of (1) variable degrees of crystallisation in different parts of the system and (2) segregation via transport and settling processes, as less dense glass fragments remain aloft at the proximal sampling location. Fracture development in crystal-bearing magmas under different shearing conditions is thought to be controlled by the magnitude of applied stress^57^ and distribution of phenocrysts as they accumulate stress and break before the interstitial melt^72,73^. Glass fragmentation generally produces complex conchoidal fracture sets, whereas crystals may break preferentially along crystallographic planes^74^ or at crystal boundaries^75^ potentially ‘liberating’ the crystal. We propose that phases with lower fracture toughness (e.g., mica, amphiboles and silicate glasses^63,76,77^) are more susceptible to fracturing during pyroclastic transport through milling and abrasion – however, crystals with low fracture toughness and strong cleavage planes, such as amphibole, may be efficiently removed during abrasion due to fracture along the cleavage planes, rather than preserved as a rind, which may explain the amphibole depletion in sample DC (Fig. 4).

### Eruptive processes at Santiaguito

It has been previously noted that abrasion results in glassy margins forming around phenocrysts in PDC deposits. We also show that an enrichment of quartz and pyroxene and depletion of Ti-magnetite at particle boundaries may also be characteristic of abrasion and milling in PDCs. It is noteworthy that, with the exception of amphibole, the results of this study show the same trends of enrichment or depletion of boundary phases for both samples (Fig. 4b), however the magnitude of the variations is greater for sample DC. This may be partly explained by the mechanics of the Caliente dome at Santiaguito, which point to similarities in ash production processes for both types of activity in dense, crystal-rich lavas. The piston-like, fault-controlled dynamics of dome deformation^27,42^ together with the generation of gas-and-ash plumes along fractures, suggest that magmatic fragmentation as well as faulting and cataclasis (abrasion along faults and particle interaction^11^) contribute to the generation of volcanic ash at Santiaguito. The combined contribution of magmatic fragmentation and cataclasis is in agreement with the vesicle-poor pyroclasts (Fig. 3a) and with the recent suggestion that fragmentation due to late thermal vesiculation modifying local stresses along active shallow faults controls the localised development of gas-and-ash explosions, which stabilises the dome structure^27^. Separate gas- and ash-rich plumes are often observed (erupted in sequence from one fracture or from adjacent fractures), where a gas-rich, ash poor plume appears first, followed by the ash-rich plume (note the greater gas-rich plume height in Fig. 1b). In this scenario, magmatic fragmentation may contribute to the ash emitted during an eruption to a much lesser degree. The range of significant fragmentation processes in such a scenario is more varied than for ash fragmented from bubbly magmas as in Strombolian^78^ and Plinian eruptions^79^, and may include (1) faulting and cataclasis, with recycling in subsequent explosions^23,25,80^; (2) magmatic fragmentation triggered by pore overpressure^54^; (3) particle interactions during transport (whether in pyroclastic density currents or dense jets^8,11,81,82^; and 4) magma-groundwater interaction^82^. A combination of these mechanisms may be characteristic of explosions in relatively dense, crystal-rich dome lavas.

Here, the observation that vulcanian explosions of ash (more enriched in interstitial glass than the ash from dome collapse) show no preferential distribution of glass or plagioclase at particle boundaries suggests that magmatic fragmentation processes at Santiaguito either do not favour fracture propagation through these phases or at their interfaces, or the fragmentation processes involved overprint one another. However, increased fractions of amphibole and pyroxene at the particle boundaries suggest the fragmentation processes may leave a characteristic fingerprint. We surmise that fragmentation of volcanic material is dependent on the physical properties of constituent crystals and glass (in addition to the physical texture), resulting in non-random fracture pathways that produce a characteristic distribution of phases at particle boundaries^46,64,65^. Such considerations are of great importance in assessing environmental and respiratory health hazards^83,84^, aviation hazards^15^ and electrostatic potential^85^ and further investigation into the role of varying fragmentation modes and conditions on ash particle surfaces is encouraged.

## Conclusions

Volcanic ash produced during a vulcanian explosion and a dome-collapse event is analysed using SEM and QEMSCAN to provide a quantitative map showing the distribution of phases within the ash particles. We use image analysis to measure the relative fraction of constituent phases at ash particle boundaries and within the bulk for all particles within size bins from 2–8 Ф. The volcanic ash produced during both eruptive styles is blocky and poorly vesicular, and the edges are generally smooth and simple. This study shows that ash particles produced during an explosion show a negligible disparity in phase distribution of the two dominant phases, plagioclase and glass, but boundaries are enriched in quartz; pyroxene and amphibole. Ash lofted from pyroclastic flows following a dome collapse also have boundaries enriched in pyroxene and quartz, but show amphibole depletion. These particles also show a higher glass fraction and less plagioclase at particle boundaries: This preferential distribution of glass along the particle margins indicates that dominant fragmentation modes in the pyroclastic flow (e.g. milling and abrasion) favour fracturing within the glass phase, while plagioclase crystals are relatively preserved or encased in a glassy rind. Repeated trends of depletion and enrichment of boundary phases in both samples, although with different magnitudes, suggest similarity in fragmentation modes during explosions and within PDCs at Santiaguito. This is supported by the observation of fault processes during eruptions at Santiaguito, and may indicate a greater contribution of ash from rupture, faulting and abrasion than previously envisaged during lava dome activity. However, the evidence from this study does not provide a clear signature for the fragmentation mechanisms during regular gas-and-ash explosions at Santiaguito and further research is required.

QEMSCAN phase maps offer the potential for rapid, high-resolution image analysis of particles and constituent phases at unprecedented sample sizes and we encourage wider adoption of automated mineralogy in the study of volcanic ash. The observed disparity in phase fractions at particle boundaries suggest that fracture locations are dependent upon the fragmentation mechanism and the constituent phases; this in turn influences the surface mineralogy and chemistry of the ash population. Measurement of the phase distribution in volcanic ash particles using the analytical method presented here can provide a powerful tool to constrain fragmentation mechanisms and understand the effect of varying eruptive activity on a range of hazards. Further research into the fingerprint of fragmentation using a range of geometric, statistical and analytical methods is required to improve our understanding of the hazards presented by volcanic ash during a range of eruptive scenarios.

## Supplementary information

Supplementary Information

## Data Availability

All data generated or analysed during this study are included in this published article (and its Supplementary Information files). Any working documents (e.g. MS Excel files) are available from the corresponding author by request.
