# Supplementary material for: Phase partitioning during fragmentation revealed by QEMSCAN Particle Mineralogical Analysis of volcanic ash
Source: Sci Rep. 2019 Jan 15;9:126. doi: 10.1038/s41598-018-36857-4 (PMC6333781; doi:10.1038/s41598-018-36857-4)
Supplement: Supplementary file 1 — Supplementary Information [file 41598_2018_36857_MOESM1_ESM.pdf]

Phase partitioning during fragmentation revealed by QEMSCAN particle mineralogical analysis of volcanic ash

A. J. Hornby[1,4], Y. Lavallée[1], J. E. Kendrick[1], G. Rollinson[2], A. Butcher[3], S. Clesham[1], U. Kueppers[4], C. Cimarelli[4], G. Chigna[5]

[1]Department of Earth, Ocean and Ecological Sciences, University of Liverpool, 4 Brownlow Street, Liverpool L69 3GP, UK

[2]Camborne School of Mines, CEMPS, University of Exeter, Penryn Campus, Treliever Road, Penryn, Cornwall, TR10 9EZ, UK

[3]Geological Survey of Finland, Espoo, FI-02151, Finland

[4]Department of Earth and Environmental Sciences, Ludwig-Maximilians-Universität München, Theresienstrasse 41/III, 80333 Munich, Germany

[5]Instituto Nacional de Sismologia, Vulcanologia, Meteorologia e Hydrologia (INSIVUMEH), 7a Avenue 14-57, Zone 13, Guatemala City, Guatemala

Corresponding author: Adrian Hornby (a.hornby@lmu.de)

Contents

Supplementary method and results

Supplementary Table 1 and 2

Supplementary Figures 1 to 5

Introduction

The unaltered QEMSCAN particle mineralogical analysis images are provided in Supplementary Figures 1-4, together with the basic criteria for phase assignment in Supplementary Table 1. These are presented here as tiff images to provide a complete record of the data used in this study. A further particle size distribution analysis is presented that does not have a major bearing on the conclusions of the study but is added for completeness and clarity. These include the particle size distribution (PSD) of each sample (Supplementary Figure 5) together with statistical analysis of the PSDs (Supplementary Table 2). Additional method to measure and analyse the PSDs is included, together with a description of the PSD results.

Supplementary method and results

Particle size analyses were performed in a Coulter LS230 laser diffraction particle size analyzer, following the methods described in Blott et al.[1]. Fresh ash samples were mixed prior to selection

of an aliquot for analysis. The aliquot was dispersed and gently mixed in a solvent prior to measurement to break down any aggregates. Coulter laser particle size analysis shows asymmetric PSD for both ash samples (Supplementary Figure 6). The ash from the vulcanian event (VE) has a minimum particle size of ~0.4 μm, a mode of 30 μm, and a maximum particle size of 800 μm; the ash from the dome collapse event (DC) has the same minimum particle size, but a mode of 50 μm and a maximum particle size of 150 μm. Closer examination reveals that the DC sample is slightly enriched in the finest particle sizes (<5 μm) and depleted in particle sizes >100 μm. The PSD curve for sample DC decreases smoothly from the modal peak whereas the curve for VE appears relatively convoluted at particle sizes >150 μm. Both samples show a similar flattened tail at the finest sizes, indicating an enrichment of fine ash particles. Statistical analysis of the PSDs, calculated using the logarithmic Folk and Ward method[2] within the GRADISTAT Excel package[3] are shown in Supplementary Table 2.

Supplementary Table 1: QEMSCAN identification criteria and range of possible mineral matches within each mineral category, applied during particle mineralogical analysis (PMA). Manual checks and comparisons with external data, including EPMA measurements of interstitial glass, were made before phase assignment was finalized.

Mineral Category	Mineral Description
Plagioclase feldspar	Plagioclase feldspars: phases with Ca,Al,Si,O.
Glass	Any phase with K,Al,Si,O (including orthoclase/microcline/sanidine) and Na,Al,Si,O (albite).
Pyroxene	Any phase with Mg,Fe,Si, such as olivine, serpentine group and orthopyroxenes
Amphibole	Any phase with Ca,Mg,Fe,Si, (with or without Al) such as hornblende, tremolite, augite, actinolite.
Ti Magnetite	Any phase with Fe,O and low Ti. May include other Fe oxides such as magnetite, hematite, goethite.
Quartz	Quartz and other silica minerals/ polymorphs.
Ilmenite	Any phase with Fe,Ti,O.
Apatite	Any phase with Ca,P,O.
Others	Any other mineral not included above. These may include Fe sulphides, Cu-Fe-sulphides, kaolinite/ halloysite/ dickite, chlorite, and phases with Fe-Al-Si (±Mg).
Background	All resin related/edge effects.

Supplementary Table 2: Particle size distribution statistics for sample VE and DC, shown in Supplementary Fig. 5, calculated using the logarithmic Folk and Ward method

	VE	DC
Mean	5.18	5.30
Median (MdΦ)	5.05	4.95
Sorting (δ)	1.83	1.81
Skewness	0.46	0.77
Kurtosis	3.24	3.01

Supplementary Figure 1. QEMSCAN PMA image output for sample VE (polished section) at 1 μm resolution (1 pixel = 1 μm^2). Any colours not included in the key represent minor phases (altogether comprising <2% total phase area) and are included in 'Others' in all analyses.

Supplementary Figure 2. QEMSCAN PMA image output for sample VE (polished section) at 2.39 μm resolution (1 pixel = 5.71 μm^2). Any colours not included in the key represent minor phases (altogether comprising <2% total phase area) and are included in 'Others' in all analyses.

Supplementary Figure 3. QEMSCAN PMA image output for sample DC (polished section) at 1 μm resolution (1 pixel = 1 μm^2). Any colours not included in the key represent minor phases (altogether comprising <2% total phase area) and are included in 'Others' in all analyses.

Supplementary Figure 4. QEMSCAN PMA image output for sample DC (polished section) at 2.39 μm resolution (1 pixel = 5.71 μm^2). Any colours not included in the key represent minor phases (altogether comprising <2% total phase area) and are included in 'Others' in all analyses.

Supplementary Figure 5. The particle size distribution for samples VE and DC measured by a Coulter L230 laser particle analyser, with sample VE in red and DC in blue.

References

1. Blott, S. J., Croft, D. J., Pye, K., Saye, S. E. & Wilson, H. E. Particle size analysis by laser diffraction. *Geol. Soc. London, Spec. Publ.* **232,** 63–73 (2004).
2. Folk, R. L. & Ward, W. C. Brazos River bar [Texas]; a study in the significance of grain size parameters. *J. Sediment. Res.* **27,** 3–26 (1957).
3. Blott, S. J. & Pye, K. Gradistat: A Grain Size Distribution and Statistics Package for the Analysis of Unconcolidated Sediments. *Earth Surf. Process. Landforms* **26,** 1237–1248 (2001).

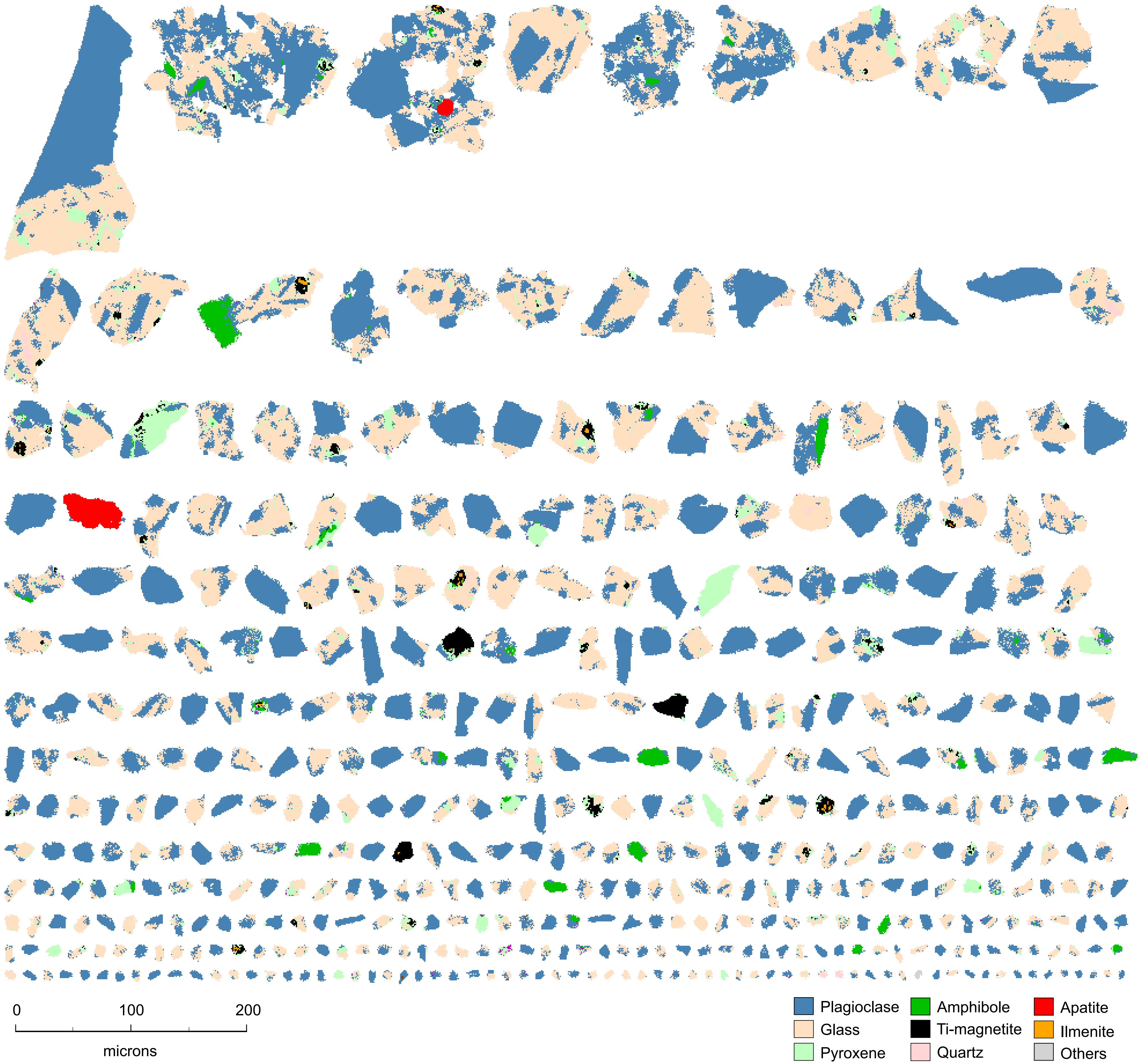
0
100
200
microns
Plagioclase
Glass
Pyroxene
Amphibole
Ti-magnetite
Quartz
Apatite
Ilmenite
Others

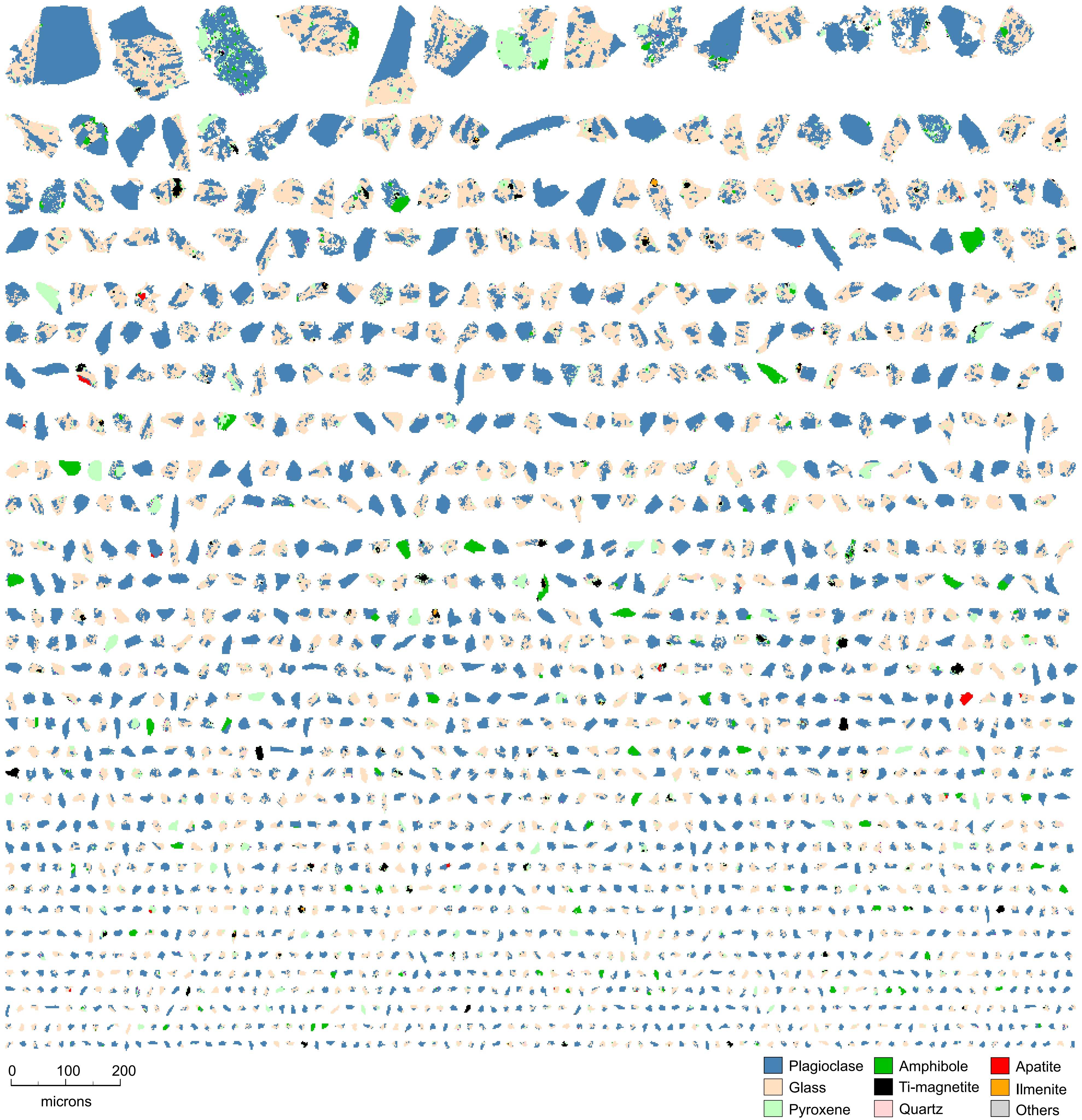
0
100
200
microns
Plagioclase
Glass
Pyroxene
Amphibole
Ti-magnetite
Quartz
Apatite
Ilmenite
Others

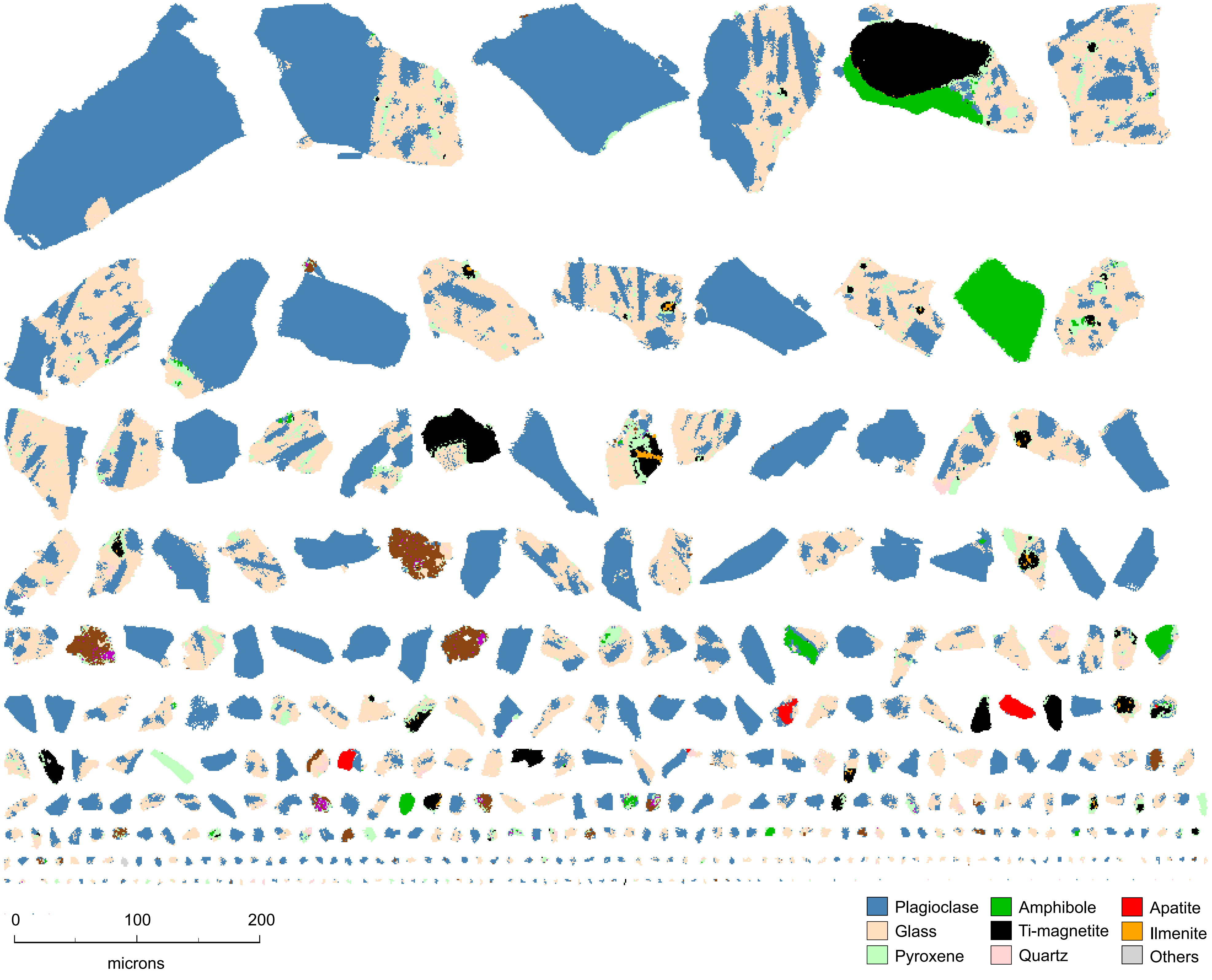
0
100
200
microns
Plagioclase
Glass
Pyroxene
Amphibole
Ti-magnetite
Quartz
Apatite
Ilmenite
Others

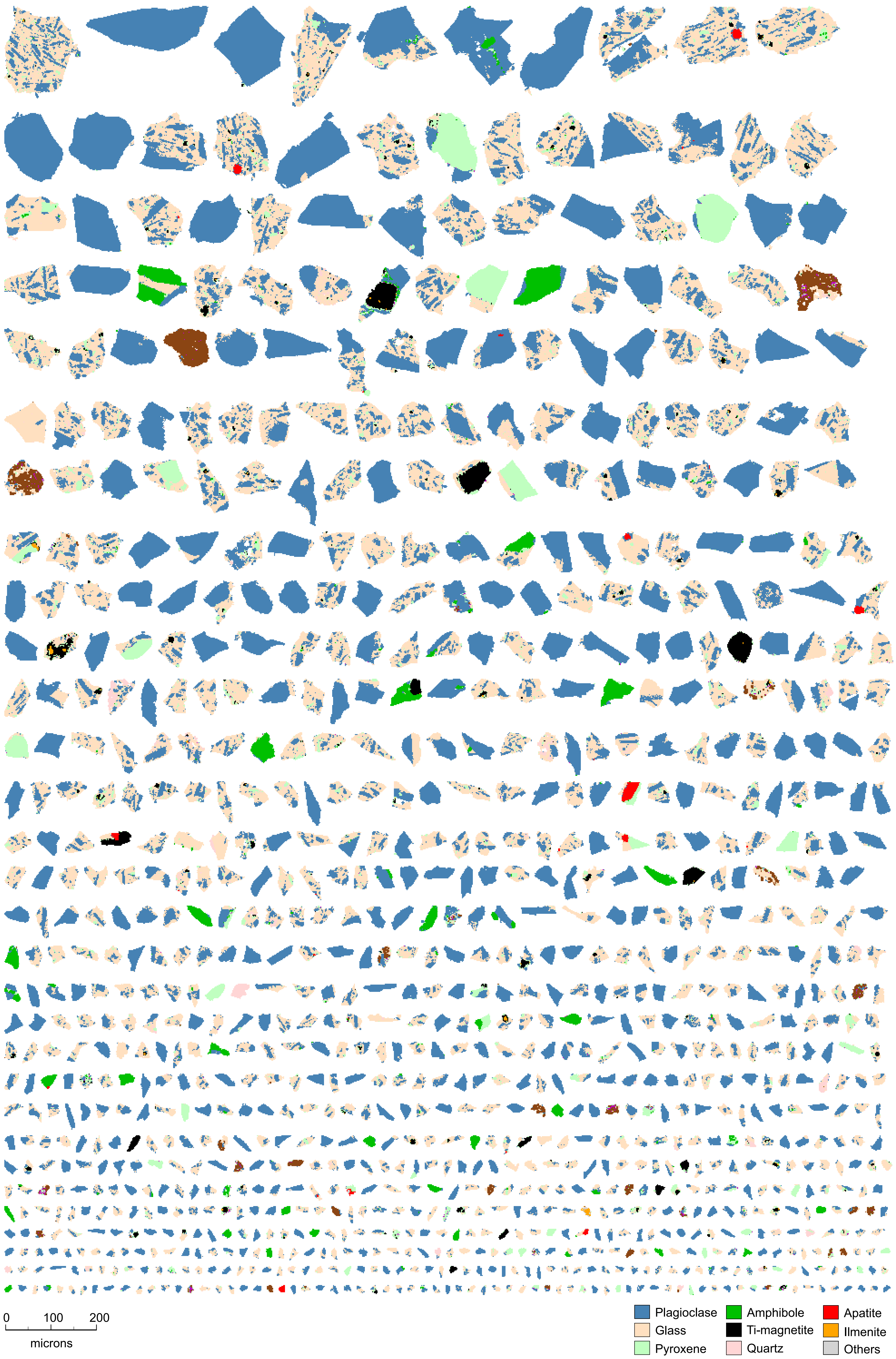
0
100
200
microns
Plagioclase
Glass
Pyroxene
Amphibole
Ti-magnetite
Quartz
Apatite
Ilmenite
Others

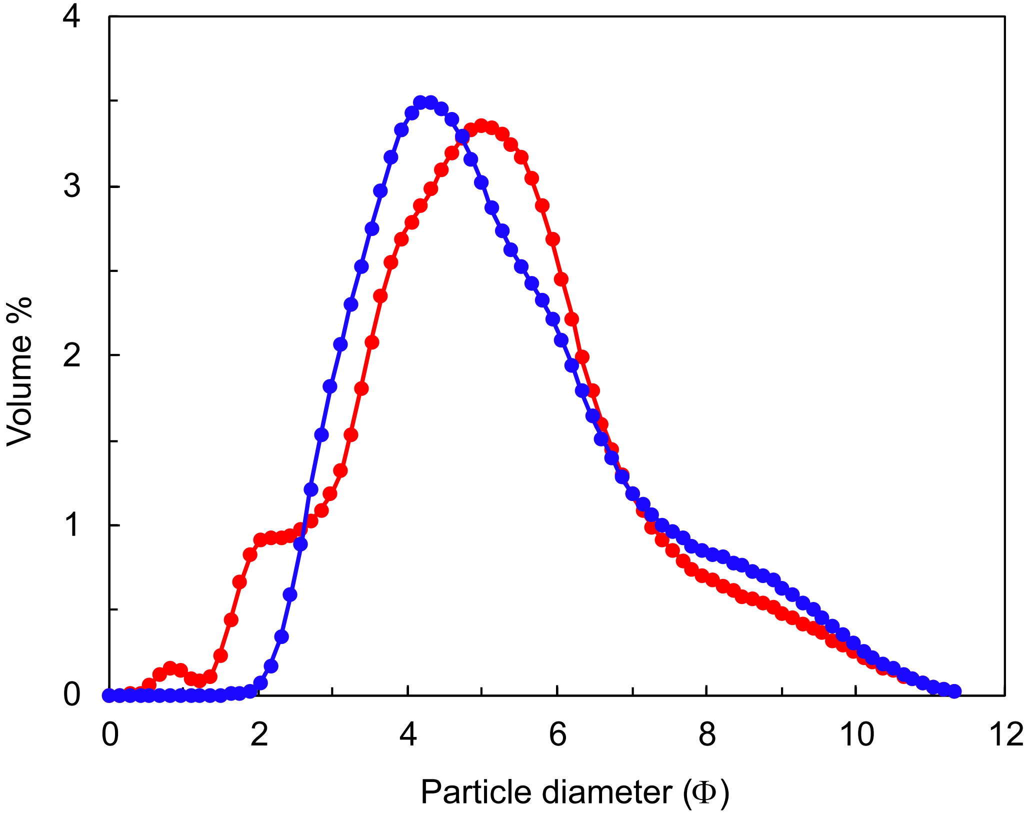

4
3
2
1
0
Volume %
0
2
4
6
8
10
12
Particle diameter (Φ)
